# Facilitators and barriers associated with breastfeeding among mothers attending primary healthcare facilities in Mpumalanga, South Africa

**DOI:** 10.3389/fnut.2023.1062817

**Published:** 2023-03-14

**Authors:** Ethel Sekori Seabela, Perpetua Modjadji, Kebogile Elizabeth Mokwena

**Affiliations:** ^1^Department of Public Health, School of Health Care Sciences, Sefako Makgatho Health Sciences University, Ga-Rankuwa, Pretoria, South Africa; ^2^Non-Communicable Diseases Research Unit, South African Medical Research Council, Cape Town, South Africa

**Keywords:** breastfeeding, facilitators, barriers, qualitative research, primary health facilities, South Africa

## Abstract

**Introduction:**

Despite the health benefits of breastfeeding for both the mother and the child, early cessation of breastfeeding remains a public health problem in South Africa, attributed to contextual barriers and facilitators. Within the context of Mpumalanga province, which is characterized by low breastfeeding rates and high infant mortality rates in children under 5 years, we explored the facilitators and barriers to breastfeeding among mothers attending the three primary health facilities in Ermelo.

**Methods:**

Using a semi-structured interview guide suggested by the socio-ecological model, three focus group discussions and 12 in-depth interviews were conducted among mothers selected using a purposive sampling. Transcripts from audiotaped and transcribed verbatim interviews were assessed through thematic analysis using NVivo version 10.

**Results:**

Mothers were aged between 18 and 42 years and from poor sociodemographic backgrounds. At the individual level, mothers valued breastfeeding facilitated by their commitment, maintaining it, eating healthy foods, and having sufficient breast milk. However, returning to work, insufficient breast milk, misconceptions about breastfeeding, and interference with social life were the barriers for mothers to breastfeed continuously. At the interpersonal level, the family was identified as the main form of support to breastfeeding mothers; however, family interference was also identified as a barrier. At the community level, mothers shared some family beliefs and practices but were still split between societal and cultural norms and traditional beliefs as facilitators or barriers to breastfeeding. At the organizational level, most mothers valued the support provided by healthcare workers on childcare and techniques for breastfeeding at the health facilities. They did however articulate concerns on the miscommunication some healthcare workers offered regarding breastfeeding, which negatively influenced their infant feeding practices.

**Discussion:**

Intervention efforts should focus on behaviour change to educate and equip mothers to overcome the barriers that are within their control. Such interventions should further focus on family-centered education and strengthening the proficiency of healthcare workers on advising breastfeeding mothers.

## Introduction

Breastfeeding is a behavior that relates to the relationship between the mother and the child (1). The World Health Organization recommends early initiation of breastfeeding, exclusive breastfeeding, and timely introduction of complementary feeding and continued breastfeeding for up to 2 years or beyond (2). In sub-Saharan Africa (SSA), South Asia, and parts of Latin America, the rate of breastfeeding has been estimated at 12 months, on average, with suboptimal exclusive breastfeeding among infants younger than 6 months in sub-Saharan Africa (3). Countries in East and South Africa have lower rates of continued breastfeeding, on average, but higher rates of exclusive breastfeeding than West Africa (4). Initiation and continuation of breastfeeding are closely related to the sociodemographic status of the mother (5–7), while successful breastfeeding is influenced by the mother's subjective norms regarding infant feeding, together with previous breastfeeding experience, her age, knowledge, attitudes, beliefs, and expectations (1, 8). The literature documents many advantages of breastfeeding for infants and their mothers (9, 10), including health, nutritional, and psychological benefits (3, 10). However, shorter breastfeeding duration predisposes infants to poor child survival and the risk of infectious and chronic diseases, including diarrhea and respiratory tract infections (3, 11), and mothers, to increased risk of breast and ovarian cancer, type 2 diabetes, and hypertension (12).

Breastfeeding can be negatively (i.e., barriers) or positively (facilitators) influenced at various levels from individual, interpersonal, community, and organizational to policy levels (13). In the sequence of these levels, low self-efficacy, lack of partner support, community stigma, hospital formula samples, and lack of protective laws hinder (i.e., negative) breastfeeding (14–16). On the contrary, the positive elements of the aforementioned levels can facilitate breastfeeding (8, 17–19). Documented influences on breastfeeding around cultural traits may be harmful, harmless, or beneficial to the optimal breastfeeding practices (20) and implicated in the early cessation of breastfeeding (21–23). Early cessation of breastfeeding has been attributed to the lack of support from family members or healthcare workers (HCWs), peer pressure, mothers' body image, the role of women in the reproduction process, and pressure to use artificial feeding (17, 24, 25). Precisely, culture affects the role of women regarding breastfeeding and may create doubts about the mother's natural ability to feed the baby, resulting in early cessation of breastfeeding (15).

In South Africa, research on breastfeeding practices has reported that 75–95% of mothers initiate breastfeeding within an hour after birth (75–95%) (18), with suboptimal rates of exclusive breastfeeding reported (19), especially among vulnerable populations, including those living in poor conditions (26). Significant disparities exist within breastfeeding across income, the mother's educational status, and race (27). A breastfeeding paradox in some contexts shows that infants from low-income households are the least likely to receive optimal breastfeeding and most likely to be food-insecure (28). Early cessation of breastfeeding and mixed feeding are common among South African mothers, including the addition of other liquids and complementary feeds in the first 6 months of a child's life being a norm (28, 29). Among children aged 0–5 months, 25.2% were not breastfed at all, 11.4% were fed breast milk and other milk, and 17.6% were given complementary feeds (30). On a broader picture, socio-cultural beliefs, illiteracy of mothers, home delivery, cracked nipples, milk insufficiency, and breast engorgement of mothers were found to be major barriers and factors that influence breastfeeding practice in terms of initiation, exclusivity, and duration in SSA, especially in West Africa (31).

The key facilitators of breastfeeding, especially exclusive breastfeeding, are summarized as breastfeeding knowledge, high levels of self-confidence, the presence of a spouse/partner who assists with chores, family support, and nurses who provided breastfeeding information (32, 33), but barriers to breastfeeding are enormous (34–36). From the few provinces in South Africa, such as Kwa-Zulu Natal, North West, and Western Cape, barriers to breastfeeding have been reported as misconceptions about breastfeeding, lack of knowledge, desire for social acceptance, pressure to maintain an ideal body shape, fuelled negative attitudes, insufficient milk, and reduced breast milk production (34–36). In their systematic review, Sokan-Adeaga and colleagues (31) have identified high socioeconomic status, delivery at the health facility, knowledge of mothers, and expensive infant formula as major facilitators prevalent in SSA, mostly citing Nigeria and Ghana. Studies in Kenya from different contexts in Nairobi (37), Nyando (38), and Offa (39) have identified the following various factors as potential determinants of breastfeeding, especially exclusive breastfeeding: socioeconomic, demographic, maternal, socio-cultural, social, and psychosocial support factors.

Despite the importance of breastfeeding to the health of mother and child, early cessation of breastfeeding remains a societal concern, attributed to contextual/environmental barriers and facilitators. Facilitators and barriers to breastfeeding are well-established in other provinces in South Africa (34–36), but data on the totality of breastfeeding pressing issues are scarce in most regions in Mpumalanga province according to Nieuwoudt and colleagues (40). In addition, previous studies addressing the challenges associated with breastfeeding have been mostly quantitative, with only a few using a qualitative approach, including in South Africa (30, 41). Therefore, within the context of Mpumalanga province, characterized by low breastfeeding rates (42, 43) and high infant mortality rates in children under 5 years (44), we explored the facilitators and barriers to breastfeeding by interviewing using a qualitative approach among mothers attending the primary health facilities in Ermelo. Improving nutrition and preventing child mortality are United Nations' goals to be met by 2023, and South Africa has a golden opportunity to scale up breastfeeding through multisectoral approaches, investment, and systemic change (4, 45, 46).

## Materials and methods

### Study design and theory

This study used a qualitative descriptive design to explore contextual facilitators and barriers to breastfeeding among mothers in primary health facilities in Mpumalanga, South Africa. The study was conducted between May 2019 and October 2019 and adhered to the Consolidated Criteria for Reporting Qualitative Research (COREQ), a 32-item checklist for interviews and focus groups (47). A socio-ecological model (SEM) was used to describe the facilitators and barriers to breastfeeding among mothers (48). Using this framework, research has shown that individual factors (i.e., low self-efficacy), interpersonal (i.e., lack of partner support), community (i.e., community stigma), organizational (i.e., hospital formula samples), and policy (mainly lack of protective laws) hinder breastfeeding (48). On the contrary, the positive side of the aforementioned barriers facilitates breastfeeding on each level, such as high self-efficacy (individual), supportive family and friends (interpersonal), access to community resources (community), in-hospital education (organizational), and workplace protections (policy) (48). Furthermore, there are individuals within a breastfeeding women's microsystem that can influence each level of the SEM, such as family and/or friends, childcare providers, and HCWs (48).

### Study setting and population

The study was conducted in a small town, located in the Mpumalanga province of South Africa called Ermelo, in the Msukaligwa local municipality of Gert Sibande district. At the time of data collection, this area had three primary health facilities and a regional hospital providing healthcare services to the majority of the residents. Data were collected in all three primary health facilities. The population of Ermelo is estimated to be 96,219, close to 60% of the total Msukaligwa population of 164,608, with most being Black people and mainly speaking isiZulu.

Purposive sampling was used to select mothers of children under the age of 2 years from an approximated number of mothers (i.e., 30 to 50/week) attending childcare services at the primary health facilities. With the help of nurses, mothers were recruited by the principal researcher while waiting to be attended to and were informed about the purpose of the study. Participants who showed interest and met the inclusion criteria were invited to participate in the study. During recruitment in the health facilities, information leaflets written in isiZulu to explain the overall aim, objectives, and procedures were distributed to the mothers, while further details were explained by the researcher and the research assistants. Mothers were eligible to participate in the study if they were at least 18 years old and able to provide written informed consent, had a child aged under 2 years, and had ever breastfed, or were breastfeeding at the time of the study. Mothers were recruited while in the queues for child services, and those who met the inclusion criteria were identified and approached with the help of the nursing staff at the respective facilities. The researcher and the research assistant explained the purpose of the study in detail and those who volunteered to participate were requested to meet the researcher to arrange their participation in focus group discussions (FGDs) or individual in-depth interviews (IDIs).

### Data collection

A semi-structured interview guide, informed by SEM, was developed by the research team focusing on mothers' views in relation to the various levels (individual, interpersonal, community, and organizational [i.e., HCWs]) that might have facilitated or hindered breastfeeding (48). The interview guide was developed in English and translated into isiZulu. The guide consisted of open-ended questions that addressed issues about the facilitators and barriers to breastfeeding. The questions were modified as the data collection proceeded, and during interviews, follow-up questions and predefined probes were asked in response to the responses given by the mothers. During interviews, follow-up questions and probes were asked to seek clarity or further explore responses given by the mothers. The study used a combination of IDIs and FGDs to produce in-depth views of mothers on the facilitators and barriers to breastfeeding.

The sample size consisted of 30 mothers of whom 18 participated in three FGDs consisting of six members per group, and 12 participated in IDIs, and the sample size was determined by data saturation. Data saturation implies that no new information could be obtained during interviews on a question level, and entirely from the tool collectively to replicate the study, and when further coding was no longer feasible, as we kept getting repeat/identical information (49, 50). The literature acknowledges three to six FGDs and 10 to 15 IDIs suitable for achieving data saturation on a studied phenomenon (51–53). Prior to conducting and audiotaping interviews, consent to audiotape the discussion was obtained from the mothers, and pseudonyms were used for each participant. Each IDI lasted between 30 and 35 min, while FGDs took ~1 h. Both FGDs and IDIs were conducted in a private room in the facilities at separate times during the days of data collection without interfering with the daily running of the facilities, with a moderator and a note taker. Sociodemographic data on personal and household information, as well as the obstetric history, were taken at the end of each FGD and IDI using an adapted short demographic tool (54–57). The IDIs preceded the FGDs to avoid the influence of FGDs on individual interviews and helped the moderator to draw focus on possible discussion in FGDs (50). Triangulation, peer debriefing sessions, and the use of a local language during interviews were used to ensure trustworthiness, including taking field and interview notes. After the interviews, mothers were given refreshments.

### Data management and analysis

Using Braun's steps for thematic analysis (58), transcripts were read by the researchers to familiarize and immerse themselves with the data, which was followed by generating initial codes from the data using manual coding. Data were analyzed using NVivo 10 (59). All transcripts were transcribed verbatim by an experienced transcriptionist, translated into English, and later reviewed by the principal researcher to ensure accuracy and that there was no loss of meaning. Authors familiarized and immersed themselves with the depth and breadth of the content. The codebook was developed by reviewing the themes, refining them, and naming them. A rigorous process was ensured to define and reach a consensus on the emerging themes and subthemes. The themes were given definitions that determined the essence of what each theme was about and determined what aspect of the data each theme captured. Bracketing was maintained throughout the data analysis to reduce inherent biases. Once the codebook had been developed, consensus about the themes was reached. The findings are presented in themes and quotations that reflect the facilitators and barriers to breastfeeding among mothers. Data showing the process, records, and findings were kept as an audit trail (50).

### Ethical considerations

The permission to conduct the study was granted by the Sefako Makgatho Health Sciences University Research and Ethics Committee (SMUREC) (SMUREC/H/23/2019: PG), which also granted ethical approval. Further permissions to conduct the study were obtained from the Mpumalanga Province Department of Health (Reference number MP_201905_004), South Africa, and from the managers of the three primary healthcare facilities. All mothers gave consent to participate in the study.

## Results and findings

### The characteristics of mothers and children

Table 1 shows the characteristics of mothers and their children. The mean age of mothers was 27 ± 6 years, ranging from 18 to 42 years, while the mean age of children was 9 ± 7 months, with 70% of them under 1 year. Most of the mothers were single, unemployed, had no tertiary education, and were from households with a monthly income of below R5,000/month (≈$282,20). Poor obstetric history was observed in terms of unplanned pregnancy, late or no attendance of antenatal care, cesarean mode of delivery, and living with HIV. The mothers included in this study gave birth to boys, born last, and all of them were HIV-negative.

**Table 1:** Characteristics of the mothers and children.

**Variables**	**Categories**	***n***	**%**
Age	≤30 years	21	70
	>30 years	9	30
Marital status	Single	25	83
	Married	2	7
	Cohabiting	3	10
Education	Primary	10	33
	Secondary	1	3
	Completed matric	17	57
	Tertiary	2	7
Employment status	Employed	8	27
	Unemployed	22	73
Household income/month	< R5000 (< $282,20)	29	97
	>R5000 (>$282,20)	1	3
Maternal HIV status	Positive	12	40
	Negative	18	60
Pregnancy planned	No	20	67
	Yes	10	33
Number of pregnancies	1	6	20
	2	9	30
	≥3	15	50
Parity	1	8	27
	2	8	27
	≥3	14	46
Attended ANC	No	4	13
	Yes	26	87
Time attended ANC	≤3 months	14	47
	>3 months	13	4
	Never attended	4	13
Delivery mode	Normal	21	70
	Cesarean	9	30
Child sex	Boy	20	67
	Girl	10	33
Child age	< 1 year	21	70
	>1 year	9	30
Childbirth order	Last	23	77
	Only	7	23
Child HIV status	Negative	30	100
	Positive	0	0

### Emergent themes

**Figure 1:**
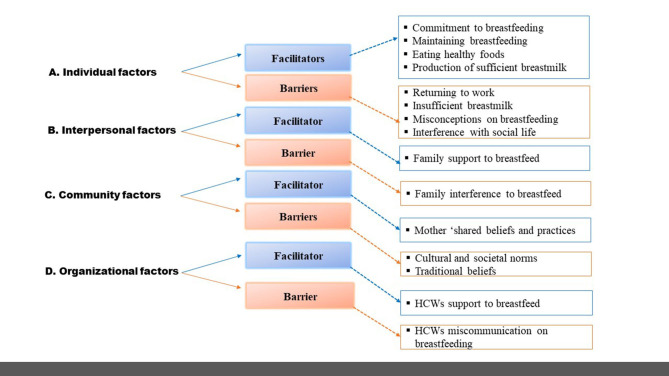
Emergent themes from IDIs and FGDs.

In Figure 1, the themes were identified at four levels of SEM, which are individual, interpersonal, community, and organization, related to facilitators and barriers to breastfeeding identified from the interviews. Individual factors facilitating breastfeeding were commitment to breastfeeding, maintaining breastfeeding, eating healthy foods, and production of sufficient breast milk, while the barriers were returning to work, insufficient breast milk, misconceptions about breastfeeding, and interference with social life. Interpersonal factors were characterized by family support to breastfeed (facilitator) and family interference to breastfeed (barrier), while community factors included the mother's adopted societal cultural beliefs and practices (facilitator), cultural and societal norms, and traditional beliefs (community). The organizational factors took into consideration the interaction of healthcare workers with mothers while at the facilities, as well as HCWs support to breastfeed (facilitator), and HCWs miscommunication about breastfeeding (barrier).

#### First level—Individual factors

In the context of this study, facilitators of breastfeeding reflect on the responses that mention factors that enhanced breastfeeding, while the barriers to breastfeeding reflect on the responses that mention any situation that makes breastfeeding practice difficult.

On an individual level, commitment to breastfeeding, maintaining breastfeeding, eating healthy foods, and the production of sufficient breast milk were categorized to show the high self-efficacy of a mother regarding breastfeeding. Some mothers reported that it was their choice to breastfeed (i.e., commitment), while others expressed breast milk for baby feeding in their absence and continued to breastfeed under the circumstances (i.e., maintaining breastfeeding). The commitment to breastfeeding was also observed through eating healthy foods to promote the production of sufficient breast milk (i.e., facilitators).

- Commitment to breastfeeding—refers to the dedication and sacrifices mothers made to ensure that they continued to breastfeed. From the perspective of some mothers, breastfeeding is a commitment, to the point that one mother mentioned that she made a choice prior to delivery that she will breastfeed, while one had to stop working to engage in breastfeeding. For instance, mothers said:

“*I have made peace before the child was born that I must breastfeed the child” (IDI, Participant 12)*.

“W*ith this child that I recently gave birth to, I can say that because I was breastfeeding the child exclusively, I had to stay with her so that when she needed the breast she could feed, I even had to stop working” (IDI, Participant 3)*.

- Maintaining breastfeeding—refers to responses that mention efforts to continue breastfeeding despite any circumstances. Mothers talked about the significance of continuing to breastfeed, to the point of producing milk for a child to feed in their absence while they were visiting town or frequenting between home and the workplace:

“*When I had to go to town and had to leave the child, I had to express milk a leave it behind*” *(IDI, Participant 12)*.

“*I once tried to give the child formula milk just after I returned from the hospital. Now I am not giving it, I was doing it so that when I return to work then the child will feed on the formula. But then now when I go to work, I leave him breastfed, and I can come back again at 10 am, then again at 1pm because I am working close to home” (*FGD 3, Participant 3).

- Eating healthy foods—reflects on responses that mention certain foods significantly linked to producing sufficient breast milk. Mothers explained how they consumed some foods that are known to boost breast milk production, like salted ground nuts, soft porridge, water and tea, and maize-based foods. Mothers said:

“…*one must eat healthy food so that you have enough breastmilk…, like soft porridge, drink water and tea, eat the food that is maize based” (IDI, Participant 6)*.

“W*hen I gave birth to my baby I had little milk, so my friends and family had to bring me those salted ground nuts, then I could see that after eating them my breastmilk started leaking, even when I was just sitting, it was a proof that ground nuts were working, in <30 mins the milk was already there…, just like a miracle” (IDI, participant 10)*.

Individual barriers to breastfeeding typically involved returning to work, insufficient breast milk, misconceptions about breastfeeding, and interference with social life. Having to return to work was the most concerning thing for mothers because it led to early cessation of breastfeeding. This was also due to insufficient breast milk, which some mothers referred to as white water, while others felt that a child cannot survive only on breast milk. Hence, mothers introduced foods, and some used milk formulas. There were misconceptions about breastfeeding from mothers, some claiming that breast milk causes constipation, while others considered breastfeeding practice necessary for mothers living with HIV and associated with the stigma, which was concerning. More interesting is the fact that breastfeeding was hindering them to socialize or look slim. One mother mentioned the disturbance of having to take a child with her if she/he is breastfeeding to visit her partner for sexual relations and wanting to lose weight.

- Returning to work—any response indicating a return to work that hindered or led to the cessation of breastfeeding. Mothers expressed having to return to work, which made breastfeeding practice difficult. They said:

“*Yes, I have breastfed both children, but I have[sic] stopped when they were 4 months because of work, working overtime, and shift” (IDI, Participant 5)*.

“*Six months is too long, especially to[sic] those working mothers like me. It does not happen that they give you 6 months' maternity leave. Maybe they give you 3 months. Now, it is better to breastfeed for 3 months or maybe 2 months. Then, on the 2 month, you start to try to mix feeding so that a baby can be able to….by the time you go back to work she already knows how to eat yes[sic]” (*FGD 2, Participant 3).

- Insufficient breast milk—in this context, insufficient breast milk refers to the description of the inadequacy of breast milk to satisfy the child. Some mothers went on to explain how breast milk is not enough, and as a result that led them to stop breastfeeding. Mothers said:

“*Breastmilk is water and water does not satisfy…, it is just that it is colored, and is milk. Alone it will never satisfy the baby. You will always need to also feed the child solid food on a side”* (FGD 1, Participant 2).

“*Breastmilk alone is not good because the child does not get full sometime. The child does not get well satisfied. Breastmilk is like a letdown fluid to give the child after you have given solid food” (IDI, participant 1)*.

- Misconception about breastfeeding—refers to responses that describe a lack of understanding of breastfeeding. Mothers articulated misconceived ideas on breastfeeding practice to the point of associating breastfeeding with a child suffering from constipation, justifying that the introduction of solid foods assists in resolving the constipation problem. Mothers also misconceived that breast milk is not enough to fill the child, hence they believe that is the reason why a child is crying. As a result, complementary and mixed feeding at an early age, such as at 2 months, was the way to go. According to some mothers, this would lead to the child sleeping. Most concerning is that some mothers considered breastfeeding practice necessary for mothers living with HIV and associated with the stigma. Furthermore, some mothers' views indicated that breastfeeding leads to excessive weight loss to the point where they felt uncomfortable because they started to look sickish. For instance, mothers said:

“My child *took time to pass stools because she was constipated when she was feeding only on the breastmilk, and that would happen for maybe 2 days. There would still be no passing of stools, then I could see that she does not get satisfied then I decided to give her food” (*FGD 3, Participant 1).

“*Breastfeeding practice is different between those who have HIV and those who do not have it. Those who are not sick, we breastfeed until the child is 2 years old and those who are sick, they say they must breastfeed for 6 months then only after that they can start giving the child food[sic].”* (FGD 2, Participant 5).

- Interference with social life—reflects on responses that explain disturbances in their life regarding socializing, sexual relations, and weight loss. The viewpoints of mothers on breastfeeding interfering with their social life were more concerning to the point that one mother mentioned that she does not want to be left behind due to breastfeeding. As a result, she opted for formula. More interesting is the fact that one mother mentioned the disturbance of having to take a child with her if she is breastfeeding to visit her partner for sexual relations and wanting to lose weight. In addition, some mothers indicated that breastfeeding was interfering with their plan to lose weight because breastfeeding prevents them from dieting or consuming weight loss drinks and causes them to eat a lot.

“W*hat I have noticed from young mothers is that they don't[sic] want to breast feed because, they say a child that is breast feeding makes it difficult for you to socialize. It is difficult to be left behind, so they opt for formula so that when they want to go, they can do so with ease” (*FGD 3, Participant 4).

“*The child cries when you go away, you are no longer able to leave the child just for a little while, even when you want to visit the father for sexual relations. At least you must always go with child[sic] because the child is breastfeeding” (*FGD 2, Participant 6).

“*I can't even be on a diet, and now December is so close. So as for me of[sic] really, I can't even…like I am saying now that I can't even be on a diet, particularly because of the child, I can't just drink things for weight loss while I am breastfeeding you see. So, some of the things [weight loss] would want me to wait this 6 month[sic] to finish breastfeeding you see” (*FGD 2, Participant 6).

#### Second level—Interpersonal factors

At the interpersonal level, the family was identified as support for breastfeeding, especially from their mothers and grandmother; however, family interference was also identified as a barrier.

- Family support for breastfeeding—refers to any mention of the support mothers received for breastfeeding. Mothers mentioned that the support they received from family members, especially from their mothers and grandmothers, for caring for the child, enabling them to breastfeed and to rest, and they said:

“*I had support from my grandmother on breastfeeding the child and that whenever the baby wakes up, I must breastfeed, and also that I must feed frequently so that the child can grow” (IDI, Participant 7)*.

“*Let's say that maybe the child did not sleep at night my mother would take the child and look after the child so that I can be able to sleep for a while during the day” (IDI, Participant 8)*.

- Family interference—refers to responses that mention obstacles to breastfeeding caused by family members' involvement. Further barriers included family interference, in which grandmothers and mothers suggested that mothers must feed their children with solid foods. Similarly, mothers introduced solids foods, purity, and formula while breastfeeding, because they felt that milk was not enough.

“*Because some of us are staying with grannies, they don't understand the situation of not giving the child food before 6 months. They always say fundza [feed] the baby. Then I would feed the baby solids and give the breast too” (*FGD 2, Participant 2).

“*At home, my mother decided that it was better that I give my child food so that we can see whether he is crying or if the baby wanted food” (*FGD 3, Participant 5).

#### Third level—Community factors

At the community level, mothers shared some personal beliefs and norms they have adopted from family beliefs and practices but were still divided between societal and cultural norms and traditional beliefs as facilitators or barriers to breastfeeding. The belief systems of individuals, families, and society significantly influence the decision-making of mothers regarding infant feeding practices.

##### Mothers' shared beliefs and practices

For example, some of the mothers reported breastfeeding choice as personal but adopted the practice from their mothers who breastfed them and their siblings. Infant feeding beliefs and practices of mothers were shared from the cultural norms and traditions of elders. Mothers said:

“*I think it is a personal decision to decide whether it is good to give breast milk only or if you want to give both*.” (*IDI, Participant 11)*.

“*I think it's a belief thing…, older people believe in cultural practices…[sic], it is a belief thing, because they believe that when you breastfeed and give solids food at the same time, the child would be well, healthy, and grow well” (*FGD 3, Participant 6).

##### Cultural and societal norms

Mothers' narratives indicated that while some of them had their own individual beliefs and attitudes about breastfeeding, there were active cultural norms and traditional beliefs, which influenced them to breastfeed and/or to opt for formula feeding or mix feed. As indicated, some of the mothers who initiated breastfeeding did so because of the family culture of breastfeeding, especially from a family perspective. Mothers said:

“*As for me, yes, I choose to breastfeed because my mother breastfed me, so I just want to follow the way of the breast and breastfeed my child” (IDI, Participant 10)*.

##### Traditional beliefs influencing breastfeeding

Mothers in this study reported traditional practices that affect their efforts to breastfeed, even exclusively breastfeeding their infants. In most African societies, performing traditional rituals on babies to protect them from evil spirits is a norm. The mothers reported that their babies were given traditional medicines: [imbiza] and other concoctions for the treatment of ibala [maroon birthmark at the back of the head and neck], as well as colic and inyoni [described as loose stools similar to diarrhea]. Mothers said:

“*Just after I was discharged from the hospital maybe after 2 weeks of discharge when the child cried too much and would not stop crying, I took the child to the healer who made a cut [razor cuts on ibala, birth mark] and then gave me imbiza [traditional medicine] for the child to drink” (IDI, Participant 9)*.

“*When the child is passing out loose stools, they call it inyoni, so they make razor cut around the umbilicus and put the traditional medicine and also the child is given traditional medicine to drink” (*FGD 2, Participant 3).

Some mothers did not believe in giving their babies traditional medicines for the treatment of colic. They reported the use of over-the-counter medications, such as Lennon Products for infantile colic, to heal a child. Mothers said:

“*I don't give anything traditional; I give only Western medicine for such things, as in case[sic], the child might absorb the bad spirits, I use the Western ones like stapes drupels you see” (IDI, Participant 6)*.

“*As for us at home, we don't use cultural medicine, so actually when you get a baby, you must just breastfeed, otherwise you just buy medicines like Phillips Gripe water, if the baby is having a troubled tummy, Bascopan, you give those” (FGD 1, Participant 4)*.

#### Fourth level—Organizational factors

At the organizational level, most mothers valued the support provided at the health facilities by healthcare workers for childcare and breastfeeding techniques, but still articulated concerns about healthcare workers' miscommunication about breastfeeding, which influenced their feeding practices negatively.

##### HCWs support for breastfeeding

Mothers described breastfeeding support by mentioning how they had received support from the HCWs regarding initiating breastfeeding after giving birth, especially on breastfeeding techniques and advice on breast milk production. Some mothers went on to discuss receiving continued support for breastfeeding while they were at the primary healthcare facilities, and they said:

“*Nurses show you how to hold the breast when breastfeeding. let us say, like with me, I would see some mothers doing this (a mother is holding the breast at the nipple with two fingers). They say it is called scissor[sic], so I also did that when I breastfed and then the nurses showed me how to do it right so that when the child is breastfeeding, you do not end up closing the nose with your breast and said doing the scissor is not right and should not be used, that is all” (IDI, Participant 8)*.

“…*Like me I once asked a nurse at the clinic about the breastmilk that in my view was not enough, that it would quickly finish when the child feeds on the breast. The nurse told me what to drink to make breastmilk increase” (IDI, Participant 2)*.

##### Miscommunication on breastfeeding by HCWs

The theme include any articulation that lacked understanding or caused contradiction regarding breastfeeding messages HCWs. Mothers articulated the miscommunication they had received from HCWs on breastfeeding, explaining the concerns about the comments made by HCWs during clinic visits for childcare services, which influenced their feeding practices negatively. Some mothers said:

“*Isn't it, like for instance when you bring the baby at[sic] the clinic for weight monitoring, you find that nurses complain about the baby's weight and they ask you if you are feeding the baby well or what, meaning breastfeeding at that time. So, then you start to think on your own that there is something missing, even though they will never tell you that this baby needs some solids too” (*FGD 1, Participant 4).

“*When I was discharged from the hospital after childbirth, my child weighed 3.06 kg, but when I brought him for weight monitoring and immunization as a newborn, he had lost weight to 2.8 kg[sic]. Mm, when the nurse was busy putting the baby on the scale, she asked me questions, but this child's weight at birth is written here in the Road to Health Birth card what[sic] made him lose weight. Then when I got home, I started feeding him”* (FGD 1, Participant 6).

## Discussion

This qualitative study was undertaken to explore contextual facilitators and barriers to breastfeeding among mothers attending primary health facilities in Mpumalanga, South Africa. The study utilized the SEM approach and interviewed breastfeeding mothers to understand, mostly, their microsystem of breastfeeding facilitators and barriers (60, 61). This study further identified factors that should be considered to improve continuous breastfeeding, including exclusive breastfeeding, like other research areas of improvement (13, 41). Mothers lived in poor socioeconomic households and most were unmarried, unemployed, received minimal income, and had low tertiary education, while poor obstetric history was characterized by unplanned pregnancy, late or zero attendance at antenatal care, cesarean mode of delivery, and mothers living with HIV, similar to other studies (62, 63). Socioeconomic status is one of the most important factors associated with health and medical outcomes of women of reproductive age, and lower status is associated with poor breastfeeding (64, 65). In addition, women with lower socioeconomic status are less likely to receive prenatal care, which is associated with poor obstetric outcomes (64–66). Early cessation of breastfeeding, including low exclusive breastfeeding, and an early introduction of solid foods observed in this study are consistent with other studies in South Africa (63, 67, 68) and other African countries (69–71).

Several ways of ensuring breastfeeding were identified from mothers' interviews. For example, at the individual level, mothers saw breastfeeding as valuable, and for that reason, they endeavored to breastfeed by being committed, maintaining breastfeeding, eating healthy foods, and ensuring that there was enough breast milk. Having the desire to breastfeed was observed among mothers who mostly considered breastfeeding as valuable. Personal decisions influenced mothers' commitment to breastfeeding as they explained that it took a firm resolve to breastfeed, and few of them exclusively breastfeed their babies, as was the case in studies conducted in Malawi (71) and South Africa (30). Mothers had to produce breast milk frequently between home and the workplace to maintain breastfeeding. Maintaining breastfeeding is strongly influenced by contextual factors and maternal sociodemographic characteristics, as well as factors related to prenatal care, delivery, and the postpartum period (72). In addition, mothers believed that eating healthy foods, such as salted ground nuts, soft porridge, water and tea, and maize-based foods, enabled breastfeeding by producing sufficient breast milk, supported by other research showing that adequate maternal nutrition plays a role in facilitating breastfeeding (70, 73, 74). It is, therefore, important to have nutritionists based at health facilities, especially in rural areas, to provide advice to mothers on the specific type of food to eat in order to produce breast milk (41).

However, this study identified barriers to breastfeeding, such as returning to work, insufficient breast milk, misconceptions about breastfeeding, and interference with social life. Mothers who were employed in this study, mentioned work-related issues hindering breastfeeding, such as working overtime and shifts (i.e., workloads), as well as the inability to get maternity leave and working at a place that does not have space to accommodate babies, as reported previously (73, 75–77). Provision should be made for mothers to take their children to work or there should be flexible working terms for breastfeeding mothers, such as working from home if there is a need (41). They also communicated a state of despair and concern when the child was crying, not being able to sleep, and not satisfied with breast milk, which suggested that breast milk is not enough, similar previous reports (35, 42). The quality of breast milk was a concerning issue for mothers, who stated that milk is white water and not sufficient to fill the child's stomach, as reported previously (35, 69). This underscores the relevance of counseling and education to identify the cause of the insufficient flow of breast milk to help mothers in managing breastfeeding challenges, as suggested (41).

Further misconceptions about breastfeeding insinuated poor awareness and lack of understanding of breastfeeding among mothers, consistent with other reports (73, 78). The misconceived association of breastfeeding with a child being constipated was disturbing, including mothers claiming that breastfeeding is relevant for mothers living with HIV and leads to excessive weight loss. Most interesting viewpoints is that breastfeeding interferes with their social life, depriving them of time to socialize, visiting their partners for sexual relations, hindering weight loss, and causing them to overeat. These factors, reported prevoiusly (79, 80), could only mean that mothers saw breastfeeding as a tiring process, and these could be one of the reasons that led to the early cessation of breastfeeding (69). The implication is that if these misconceptions are not addressed, mothers may stop breastfeeding (41), which is harmful to their health and the health of their child. Therefore, programs that encourage mothers to breastfeed must consider addressing issues like these misconceptions.

Although family support was the most identified form of support at the interpersonal level in this study, the family remained the largest barrier to breastfeeding. Mothers articulated the role that grandmothers and mothers played regarding breastfeeding and childcare. According to the literature, family members such as mothers, grandmothers, and mothers-in-law, play essential roles in promoting breastfeeding (70, 81, 82). This was reported in studies in SSA, such as in Mozambique (81), Tanzania (82), Zimbabwe (70), and Nigeria (76), supporting the positive role of mothers and mothers-in-law in encouraging breastfeeding mothers (70, 76, 82). In contrast, researchers have reported that breastfeeding mothers are not able to reject messages opposing breastfeeding from family, which ultimately affects their infant feeding practices by responding to family pressure (30). Therefore, family-centered breastfeeding education support is crucial because the inclusion of significant others and extended family members in prenatal breastfeeding education has been associated with longer breastfeeding durations (16).

At the community level, mothers in this study shared some family beliefs and practices consistent with other studies (46, 83), which have reported the important role of the social and cultural contexts of women in breastfeeding. However, mothers were still split between societal and cultural norms and traditional beliefs as facilitators or barriers to breastfeeding. Predominantly, mothers and grandmothers of the breastfeeding mother are the custodians of these norms, beliefs, and systems, which interfere with breastfeeding because mothers often feel obligated to these cultural prescriptions (70, 84). The use of herbal concoctions as medicines, which is apparent in many African populations (85), was reported to treat infantile colic and prevent bad spirits among children in this study, as in other studies (30, 70), which has adverse effects and is toxic for infants (84, 86). Conforming to cultural norms and traditional beliefs contradicts the guidance of HCWs informed by scientific evidence on breastfeeding.

Finally, at the organizational level, the focus was more on the role HCWs at the health facilities play in breastfeeding immediately after childbirth. It emerged that HCWs' breastfeeding was valued by mothers, especially in terms of breastfeeding techniques and advice on breast milk production relating to diet and childcare. However, HCWs miscommunication on breastfeeding was identified as a barrier, as in studies conducted in Zimbabwe (69, 70) and other local studies (30, 34–36). When mothers visited the facilities for childcare services, the comments made by HCWs misconstrued breastfeeding practice, negatively affecting their feeding practices. There is still confusion about breastfeeding in the context of HIV in South Africa, with some HCWs encouraging the use of formula and others breast milk (87). Some HCWs have been reported to provide breastfeeding mothers with advice that is not supportive of breastfeeding, especially exclusive breastfeeding (30). Desiring to breastfeed, valuing breastfeeding, and having supportive health facilities are associated with high breastfeeding initiation rates (34). Therefore, mothers always value HCWs' practical support (88) to continuously ensure that breastfeeding services are tailored to mothers with special needs, including those who are living with HIV and from poor socio-economic backgrounds. In addition, good communication between mothers and HCWs is critical for building mothers' confidence, promoting bonding, and the participation of mothers in the care of their babies. This might have long-term benefits for the health and wellbeing of the mother and her baby (89), while misleading information from health facilities can be detrimental.

### Limitations and strengths of the study

Although all three primary facilities operating at Emerlo were used, one of the weaknesses of this study is that it was limited to only one area, the Msukaligwa local municipality of Gert Sibande district, Mpumalanga Province. The second limitation in terms of social desirability bias is acknowledged in this study given the possibility that some mothers might have withheld information, despite using a moderator who understood the field of infant feeding and constantly reminded them about the issues of confidentiality while using probes for mothers to be open regarding their breastfeeding practices and conducting interviews in their local language, isiZulu. Third, although data collection ended when data saturation was reached, the use of 12 IDIs and three FGDs might be viewed as small. Three to six FGDs are typically sufficient to achieve data saturation (57, 59), while 10 to 15 IDIs are suitable for addressing the phenomenon being studied (58). Therefore, an integration of FGDs and IDIs enriched the conceptualization and interpretation of the phenomenon studied and enhanced the trustworthiness of the findings as mothers gave in-depth and detailed information. Fourth, not allowing the expression of the frequency of the themes or ranking them in order according to their level of importance is also acknowledged as a limitation. All the emergent themes of the study are considered important regarding breastfeeding.

## Conclusion

This study suggests that there are individual, interpersonal, community, and organizational factors that influence breastfeeding among mothers in Ermelo, South Africa. These factors are shaped by the experiences of mothers, their interaction with family members, the community environment, and HCWs. At the individual level, breastfeeding is a valued behavior, and mothers were committed to breastfeeding and maintaining breastfeeding, including eating healthy foods and ensuring the production of breast milk. However, mothers were hindered by returning to work, insufficient breast milk, misconceptions about breastfeeding, and interference with social life. At the interpersonal level, the family was identified as the greatest form of support for breastfeeding. However, family interference was also identified as a barrier. At the community level, mothers shared some family beliefs and practices but they were still divided between societal and cultural norms and traditional beliefs as facilitators or barriers to breastfeeding. At the organizational level, the support of the HCWs was most valued by mothers; nonetheless, HCWs' miscommunication on breastfeeding negatively influenced their feeding practices. Intervention efforts should focus on behavior change to educate and equip mothers to overcome the barriers that are within their control. Such interventions should further focus on family-centered education and strengthening the proficiency of healthcare workers in advising breastfeeding mothers.

## Data availability statement

The raw data supporting the conclusions of this article will be made available by the authors, without undue reservation.

## Ethics statement

The studies involving human participants were reviewed and approved by Sefako Makgatho Health Sciences University Research and Ethics Committee (SMUREC/H/23/2019: PG) and received permission from the Mpumalanga Province Department of Health, South Africa (Reference Number: MP_201905_004), and from the managers of the three primary health care facilities. The patients/participants provided their written informed consent to participate in this study.

## Author contributions

ES contributed to the research concept, developed the proposal, data collection, project administration, data transcription and translation, data analysis, and the first draft of the manuscript. PM contributed to the research concept, development of the proposal, supervision, data analysis, the first draft of the manuscript, and reviewed and approved the final manuscript. KM contributed to the first draft of the manuscript, data analysis, and reviewed and approved the final manuscript. All authors contributed to the article and approved the submitted version.
